# The social regulation of threat-related attentional disengagement in highly anxious individuals

**DOI:** 10.3389/fnhum.2013.00515

**Published:** 2013-08-30

**Authors:** Erin L. Maresh, Lane Beckes, James A. Coan

**Affiliations:** Department of Psychology, University of VirginiaCharlottesville, VA, USA

**Keywords:** social regulation of emotion, trait anxiety, attentional disengagement, fMRI

## Abstract

Social support may normalize stress reactivity among highly anxious individuals, yet little research has examined anxious reactions in social contexts. We examined the role of both state and trait anxiety in the link between social support and the neural response to threat. We employed an fMRI paradigm in which participants faced the threat of electric shock under three conditions: alone, holding a stranger's hand, and holding a friend's hand. We found significant interactions between trait anxiety and threat condition in regions including the hypothalamus, putamen, precentral gyrus, and precuneus. Analyses revealed that highly trait anxious individuals were less active in each of these brain regions while alone in the scanner—a pattern that suggests the attentional disengagement associated with the perception of high intensity threats. These findings support past research suggesting that individuals high in anxiety tend to have elevated neural responses to mild or moderate threats but paradoxically lower responses to high intensity threats, suggesting a curvilinear relationship between anxiety and threat responding. We hypothesized that for highly anxious individuals, shock cues would be perceived as highly threatening while alone in the scanner, possibly due to attentional disengagement, but this perception would be mitigated if they were holding someone's hand. The disengagement seen in highly anxious people under conditions of high perceived threat may thus be alleviated by social proximity. These results suggest a role for social support in regulating emotional responses in anxious individuals, which may aid in treatment outcomes.

## Introduction

A large body of research suggests that social proximity and interaction confer benefits ranging from buffering stress to extending life (House et al., 1988; DeVries et al., 2003). These benefits may be linked to the way supportive social contact can attenuate threat responding in the brain (Coan et al., 2006b, 2013). Recent work suggests that social support may be especially important for people high in trait anxiety, as anxiety is characterized by increased reactivity to stressors (Bolger and Zuckerman, 1995; Conner et al., 2012). Still, many questions remain about how anxious people respond to perceived threats in a supportive social context. Our goal was to examine how the presence or absence of perceived social resources alters threat-related processing in the brains of highly anxious adults.

In general, high trait anxiety corresponds with increased responsiveness to stressors. This is observed in self reported anxiety (Bolger and Schilling, 1991), autonomic reactivity (Gonzalez-Bono et al., 2002), and hormonal output (Schlotz et al., 2006). Neuroimaging has also revealed increased stress-related activity in the central nervous system (Etkin et al., 2004). For example, when anticipating a shock, individuals high in trait anxiety show exaggerated activity in the dorsal anterior cingulate cortex (dACC), somatosensory cortex, motor cortex, and hippocampus, areas related to vigilance, motor preparedness, and approach/avoidance conflict (Straube et al., 2009).

On the other hand, anxiety-related traits have also been associated with *decreased* responsivity to stress. For example, Jezova et al. (2004), found that participants with high trait anxiety had lower secretions of epinephrine, norepinephrine, and prolactin during a stressful public speaking task. Similarly, lower cortisol levels upon awakening were found in participants higher in trait anxiety (Walker et al., 2011). It has been suggested that the excessive, chronic activation of the stress response that anxious people experience may eventually lead to reduced responsiveness of the hypothalamic-pituitary-adrenal (HPA) axis (McEwen, 2007).

Recent work using functional magnetic resonance imaging (fMRI) has led some to postulate that anxiety has a curvilinear relationship with threat responding (Straube et al., 2009; Drabant et al., 2011). Straube et al. observed that while strong threats yielded a positive correlation between anxiety and activity in certain brain regions, this correlation was conspicuously negative in the ventral anterior cingulate cortex (vACC), a region within the cingulate associated with the modulation of physiological arousal (Allman et al., 2001). Similarly, modulating the intensity of anticipatory anxiety during shock threat led to monotonically linear increases in activity from safety to strong threat except in participants high in neuroticism (Drabant et al., 2011), a personality trait that is strongly related to anxiety (Luteijn and Bouman, 1988). Highly neurotic participants showed a relative decrease in neural activity in the insula and the dorsolateral prefrontal cortex (DLPFC) when shifting from moderate to strong threats. The authors theorize that this decreased activity signals a switch to an avoidant processing style in highly anxious individuals when the threat becomes severe. Furthermore, these findings suggest that anxiety alters an individual's *perception* of threat, which may function in the same way as altering the threat itself.

One method of modulating the perceived intensity of a threat is through social support. Indeed, recent work reveals that handholding mitigates the neural threat response, particularly when the hand-holder is a familiar relationship partner (Coan et al., 2006b; Conner et al., 2012). Less is known about how anxiety influences the extent and direction of this relationship. We do know that strong social ties buffer against the development of anxiety disorders (Plaisier et al., 2007), but little is known about how anxiety manifests in the context of social support.

The current study was designed to explore how the provision of social support may interact with anxiety in the neural response to threat. We measured brain activity using fMRI in a sample of participants who underwent a threat-of-shock paradigm under three conditions: while alone, while holding a stranger's hand, and while holding a friend's hand (cf. Coan et al., 2006b). Additionally, we examined how both trait and state anxiety moderated the neural threat response under these conditions. Due to differing reports in the literature, we proposed two competing hypotheses: (1) According to what we term the *potentiation* model, the relationship between anxiety and threat response is simply linear. That is, people with higher anxiety will show potentiated activity in brain areas related to stress and anticipatory anxiety (Coan et al., 2006a) in response to a threatening stimulus. The potentiation model predicts that under conditions of handholding, individuals with higher anxiety will show a reduction in threat-related brain activity such that they more closely resemble less anxious participants, particularly when holding a friend's hand (vs. a stranger's; cf. Conner et al., 2012). (2) By contrast, the *vigilance/disengagement* model suggests the relationship between trait anxiety and threat response is curvilinear, with moderate threats leading to increased vigilance in threat-related brain regions and strong threats leading to a strategy of disengagement from threat stimuli—and a concomitantly diminished neural threat response. Moreover, the vigilance/disengagement model predicts that people with higher trait anxiety will show *decreased* brain activity in areas related to anticipatory anxiety when anticipating the stimulus alone, because the intensity of the threat cue will be perceived as greater in the absence of handholding. Note that the potentiation and vigilance/disengagement models result in precisely opposite predictions. The potentiation model predicts higher trait anxiety will correspond with higher threat reactivity while alone and lower threat reactivity with social support. The vigilance/disengagement model predicts higher trait anxiety will correspond with lower threat reactivity while alone and higher threat reactivity with social support. Importantly, both models assume that social support decreases the perceived intensity of a threat cue (cf. Cohen and Wills, 1985; Coan, 2008).

## Materials and methods

### Participants

Twenty-seven participants and their opposite-gendered friends were recruited via flyers or drawn from a larger longitudinal study on adolescent social development (McElhaney et al., 2006; Chango et al., 2012). These participants are also part of a larger group in which we are studying the effects of handholding across different types of relationships (marriage, cohabitating, dating, and friends). Because this is a heterosexual sample in which the participants brought in opposite-gendered romantic partners, to maintain consistency, we requested opposite-gendered friends as well. We further requested each participant bring in a friend for whom they have not had romantic feelings. Respondents were excluded if they had current or past history of psychopathology, were pregnant, or exhibited risk for incident in the fMRI environment. Of the twenty-seven participants and their friends, two dyads were removed from final analyses for being outliers according to Mahalanobis distances. The final sample of 25 participants consisted of 13 males and 12 females, ages 23–26. Ten participants identified themselves as African-American and fifteen as White on a demographics questionnaire. Each member of the pair gave informed consent and was paid $160 for his or her participation.

### Procedure

Participants were screened via telephone and scheduled for a visit to the laboratory. During the screening, participants were informed they would receive a mild electric shock that is designed to be uncomfortable but not painful. On the scheduled day, the participant came in with his or her friend and both completed a battery of questionnaires assessing personality, attachment style, relationship measures, etc. For this study, we looked at results from the State-Trait Anxiety Inventory (STAI; Spielberger, 1983) for each participant. The STAI measures both state and trait anxiety, each using a 20-item questionnaire with a 4-point Likert scale, yielding scores ranging from 20 to 80.

### Shock paradigm

Two Ag-AgCl shock electrodes were placed on the participant's right or left ankle (counterbalanced across participants). The participant entered the fMRI scanner and anatomical scans were collected. Following this, the participant underwent the handholding paradigm. Participants viewed stimuli projected onto a screen at the back of the magnet's bore via a mirror placed on the head coil, and a button box was provided for the participant to respond to stimuli. Scanning was done under three conditions (Alone, Stranger, and Friend), the order of which was counterbalanced across participants. In the Alone condition, the participant underwent the experiment alone in the scanner. In the Stranger condition, the participant underwent the experiment while holding the handing of an anonymous experimenter of the opposite gender whom the participant did not meet until the end of the experiment. In the Friend condition, the participant held the hand of the opposite-gendered friend they had brought with them. Before each condition, the participant was informed whether he or she would be holding a stranger's hand, a friend's hand, or would be alone. The handholding partner sat on a stool next to the participant, with both participant and hand holder hands resting on the bed of the scanner, allowing each person to comfortably hold hands for the duration of the task.

**Figure 1:**
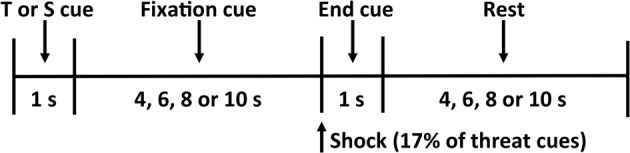
**Threat-of-shock paradigm**. Each trial consisted of a 1-s threat (T) or safety (S) cue, a 4- to 10-s fixation cross, a 1-s end cue (during which a shock was administered on 17% of the threat trials), and a 4- to 10-s rest period before the start of the next trial.

During each condition, the participant observed twelve threat (a red “X” on a black background) and twelve safety (a blue “O” on a black background) cues in a random order for a total of twenty-four trials (Figure 1). The participant was informed that the threat cue indicates he or she has a 17% chance of being shocked (i.e., two of the twelve threat cues result in a shock), and the safety cue indicates he or she is safe from shock for that trial. To increase anticipatory anxiety in our participants, we did not apply the shock before the experimental procedure and instead used a uniform shock generated by a physiological stimulator (Coulbourn Instruments, Allentown, PA) that lasted for 20 ms at 4 mA. This current was selected to provide a shock that is uncomfortable but not painful.

Each trial began with a 1-s threat or safety cue followed by an anticipation period that varied among 4, 6, 8, or 10 s, during which the participant focused on a fixation cross. A small dot indicated the end of the anticipation period, during which the shock was delivered on 17% of the threat trials. A blank screen was then presented for a 4-, 6-, 8-, or 10-s resting period, separating each trial. At the end of each condition, the participant used the button box to rate his or her subjective feelings of unpleasantness (valence) and agitation (arousal) on the 9-point pictorial Self-Assessment Manikin (SAM) scales (Bradley and Lang, 1994). We did not observe any significant effects of handholding, or indeed of state or trait anxiety, on subjective reports of valence and arousal (all *p*'s >.18).

### Image acquisition

Images were acquired using a Siemens 3.0 Tesla MAGNETOM Trio high-speed magnetic imaging device with a CP transmit/receive head coil and integrated mirror. One hundred seventy-six high-resolution T1-magnetization-prepared rapid-acquisition gradient echo slices were collected to determine the localization of function (1-mm slices, *TR* = 1900 ms, *TE* = 2.53 ms, flip angle = 9°, FOV = 250 mm, voxel size = 1 × 1 × 1 mm). Two hundred sixteen functional T2^*^-weighted Echo Planar images (EPIs) sensitive to BOLD contrast were collected per block, in volumes of twenty-eight 3.5-mm transversal echo-planar slices (1-mm slice gap) covering the whole brain (1-mm slice gap, *TR* = 2000 ms, *TE* = 40 ms, flip angle = 90°, FOV = 192 mm, matrix = 64 × 64, voxel size = 3 × 3 × 3.5 mm).

Data was preprocessed using FMRIB's Software Library (FSL) software *(Version 5.98;* *www.fmrib.ox.ac.uk/fsl**)*. Motion was corrected using FMRIB's Linear Image Registration Tool, an intra-modal correction algorithm tool (MCFLIRT; Jenkinson et al., 2002). In a separate step, we performed slice scan-time correction and a high-pass filtering cutoff point of 100 s, removing signals that were irrelevant to the stimuli. We used BET (Smith, 2002) brain extraction, which eliminated unwanted, non-brain material voxels in the fMRI data, and conducted spatial smoothing with a 5-mm full width at half minimum Gaussian kernel. Images were registered to the Montreal Neurological Institute (MNI) standard space by FLIRT (Jenkinson et al., 2002). Threat trials where participants actually received shocks were excluded from analysis due to possible movement artifacts.

### Functional regions and data analysis

Data analysis was conducted using FEAT (fMRI Expert Analysis Tool) Version 5.98 in the FSL package. For first level analysis, in order to compare the neural response to threat of shock, threat minus safety maps were created by subtracting the response to the safety cue from the response to the threat cue for each handholding condition. We chose to model the difference between the threat cue and the safety cue rather than between the threat cue and the resting period due to the ambiguity inherent in experimentally uncontrolled periods of rest (cf. Coan et al., 2006a). Moreover, only threat trials in which a shock did not occur were included for analysis in order to reduce undesirable movement artifact. For second level analysis, these data were collapsed across all three functional runs, one for each handholding condition, for each individual participant using a fixed effects model. The threat minus safe contrast from the first level was carried into the third level, where between-subjects analysis was done separately for each handholding condition, as well as on contrasts of handholding conditions (Alone minus Stranger, Alone minus Friend, Stranger minus Friend). This was accomplished using a mixed effects model with state and trait anxiety entered as covariates. All clusters were whole brain-corrected and met clusterwise thresholding of *z* > 2.3 and a corrected cluster significance level of *p* < 0.05. Anatomical labels for brain regions were identified using the Harvard-Oxford cortical and subcortical atlases. To more closely examine interactions between state and trait anxiety and handholding conditions, we extracted mean percent signal change from the hypothalamus, putamen, and multiple sites within the precuneus. All coordinates are reported in Montreal Neurological Institute (MNI) space.

## Results

### State and trait anxiety inventory results

Prior to entering the fMRI scanner, all participants completed both portions of the STAI (Spielberger, 1983). In our sample, participants scored *m* = 34.76, *SD* = 9.66 (range = 20–59) on the State portion and *m* = 32.6, *SD* = 9.08 (range = 20–50) on the Trait portion. To check for multicollinearity, we examined the correlation between state and trait anxiety. Because state and trait anxiety showed a moderate correlation in our sample (*r* = 0.42, *p* = 0.02), we tested each of them as separate predictors.

### Main effects of threat and handholding

We found main effects of threat cues minus safety cues in several areas previously found both by us and others to be active during threat anticipation (e.g., Ploghaus et al., 1999; Coan et al., 2006b). Some of these areas included the anterior cingulate cortex (ACC), posterior cingulate cortex (PCC), orbitofrontal cortex (OFC), frontal pole, angular gyrus, precentral gyrus, supramarginal gyrus, occipital cortex, caudate, putamen, pallidum, and thalamus. Main effects of handholding condition on the neural reactivity to threat in this sample have been reported elsewhere (Coan et al., 2013) and are therefore discussed only briefly here. As anticipated based on Coan et al. (2006b), threat-related (threat minus safety) activity was significantly lower in the Friend condition than in the Alone condition in the ACC, the left superior frontal gyrus, and the left supplementary motor cortex. Interestingly, threat-related activity was lower in the Stranger condition compared to the Friend condition in the left putamen.

### Trait anxiety by handholding conditions

We first identified regions of neural activity during threat that correlated with trait anxiety in each independent handholding condition (Alone, Stranger, and Friend). As described in detail below, significant negative correlations with trait anxiety were found in the Alone and Friend conditions (Table 1). No significant correlations between trait anxiety and neural activity were found in the Stranger condition.

**Table 1:** **Main effects of trait/state anxiety levels, by condition**.

**Region of peak activity**	**Cluster size (mm^3^)**	***Z*-max**	**Peak coordinates**			**Trait anx**	**State anx**
			***x***	***y***	***z***		
**ALONE**							
L precuneus	2720	3.98	−18	−62	14	−	
Precentral gyrus	2594	3.78	2	−24	56	−	
R lateral occipital cortex	1090	3.96	40	−60	22	−	
L lingual gyrus	565	3.61	−28	−50	−6	−	
L precuneus	394	3.38	−12	−58	44	−	
**FRIEND**							
L superior parietal lobule	776	4.23	−16	−58	60	−	
L frontal medial cortex	723	4.51	−14	54	−8	−	
PCC	884	4.02	0	−50	30		+
Frontal pole	535	4.59	4	62	−12		+
L superior lateral occipital cortex	517	3.88	−46	−80	24		+

*+, Indicates a positive correlation; **−**, Indicates a negative correlation*.

### Alone condition

**Figure 2:**
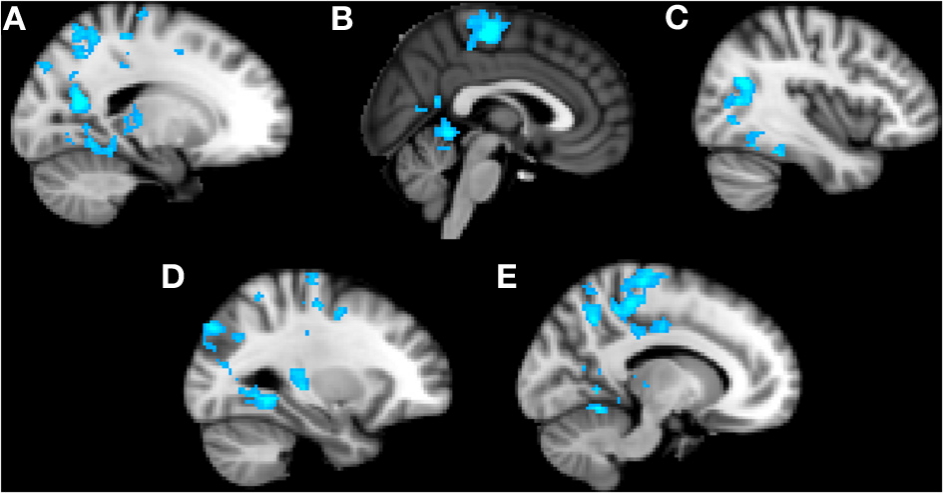
**Clusters of activity significantly correlated with trait anxiety in the Alone condition**. Blue areas indicate a negative correlation with trait anxiety. **(A)** Cluster 1, peak activation in left precuneus. **(B)** Cluster 2, peak activation in precentral gyrus. **(C)** Cluster 3, peak activation in right lateral occipital cortex. **(D)** Cluster 4, peak activation in left lingual gyrus. **(E)** Cluster 5, peak activation in left precuneus.

Trait anxiety significantly negatively correlated with brain activity in the Alone condition in five main clusters (Table 1, Figures 2A–E). The first cluster reached peak activity in the left precuneus and extended to the PCC, right temporo-occipital inferior temporal gyrus, right temporal occipital fusiform gyrus, right lingual gyrus, left cerebellum, left superior lateral occipital cortex, left insula, left inferior frontal gyrus pars opercularis, left angular gyrus, left central opercular cortex, and precentral gyrus (Figure 2A). The second cluster peaked in the precentral gyrus and extended to the PCC, ACC, supplementary motor cortex, precentral gyrus, left postcentral gyrus, left middle frontal gyrus, left anterior supramarginal gyrus (Figure 2B). The third cluster peaked in the right lateral occipital cortex and extended to the right supracalcarine cortex, right lateral occipital cortex, and right temporo-occipital middle temporal gyrus. (Figure 2C) The fourth cluster peaked in the left lingual gyrus and extended to the left posterior parahippocampal gyrus, left posterior temporal fusiform cortex, left occipital fusiform gyrus, left temporal occipital fusiform cortex, and cerebellum (Figure 2D). The fifth cluster also peaked in the left precuneus and extended into the left superior lateral occipital cortex and left superior parietal lobule (Figure 2E).

### Friend condition

**Figure 3:**
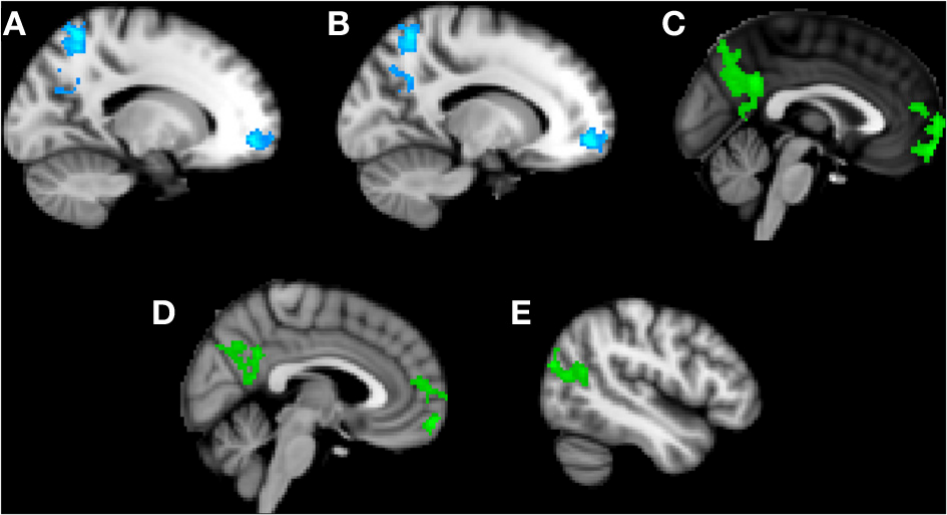
**Clusters of activity significantly correlated with trait or state anxiety in the Friend condition**. Blue areas indicate a negative correlation with trait anxiety; green areas indicate a positive correlation with state anxiety. **(A)** Cluster 1, peak activation in left superior parietal lobule. **(B)** Cluster 2, peak activation in left frontal medial cortex. **(C)** Cluster 3, peak activation in posterior cingulate cortex. **(D)** Cluster 4, peak activation in frontal pole. **(E)** Cluster 5, peak activation in left superior lateral occipital cortex.

Trait anxiety negatively correlated with brain activity in the Friend condition in two main clusters, with the first peaking in the left superior parietal lobule and extending to the PCC, left precuneus, and left superior lateral occipital cortex (Figure 3A), and the second peaking in the left frontal medial cortex and extending to the paracingulate gyrus and left frontal pole (Figure 3B).

### State anxiety by handholding conditions

Next, we identified regions of neural activity during threat that correlated with state anxiety in each independent handholding condition. Only the Friend condition yielded significant correlations (Table 1).

### Friend condition

Significant positive correlations with state anxiety were found in the Friend condition in three main clusters (Figures 3C–E). The first cluster peaked in the PCC and extended to the precuneus (Figure 3C). The second cluster peaked in the frontal pole and extended to the paracingulate (Figure 3D). The third cluster peaked in the left superior lateral occipital cortex and extended to the left angular gyrus, left inferior lateral occipital cortex, and the temporo-occipital middle temporal gyrus (Figure 3E).

### Interactions between handholding and trait anxiety

Previous research has shown that handholding by and physical proximity to close relational partners tends to attenuate threat-related neural activity (Coan et al., 2006b; Conner et al., 2012). To investigate whether levels of trait and state anxiety moderated this relationship, we employed additional contrasts (Alone minus Stranger, Alone minus Friend, Friend minus Stranger) to compare the association between anxiety and neural activity across handholding conditions. All three contrasts (Alone minus Stranger, Alone minus Friend, Friend minus Stranger) showed negative correlations between trait anxiety and threat-related brain activity (Table 2).

**Table 2:** **Interactions between handholding conditions and trait/state anxiety levels**.

**Region of peak activity**	**Cluster size (mm^3^)**	***Z*-max**	**Peak coordinates**			**Trait anx**	**State anx**
			***x***	***y***	***z***		
**ALONE − STRANGER**							
L precentral gyrus	894	3.57	−10	−28	66	−	
**ALONE − FRIEND**							
Hypothalamus	501	3.11	6	−12	−8	−	
L putamen	434	3.26	−28	−22	0	−	
Precuneus	322	3.47	2	−46	40		−
**FRIEND − STRANGER**							
Precuneus	740	3.51	0	−56	62	−	
Precuneus	2075	3.74	0	−56	14		+

*+, Indicates a positive correlation; −, Indicates a negative correlation*.

### Alone minus stranger

Subtracting brain activity correlated with trait anxiety in the Stranger condition from that in the Alone condition provides an index of brain areas that contain correlations with trait anxiety that are significantly stronger in the Alone compared to Stranger condition. The Alone minus Stranger contrast yielded one cluster of neural activity that was significantly and negatively correlated with trait anxiety. This cluster peaked in the left precentral gyrus and extended to the postcentral gyrus and precuneus. This indicated that for those with lower levels of trait anxiety, threat-related brain activity was higher in the Alone compared to Stranger condition, or, conversely, for those with higher levels of trait anxiety, brain activity during the threat cues contrasted with the safety cues was decreased in the Alone relative to the Stranger condition.

### Alone minus friend

**Figure 4:**
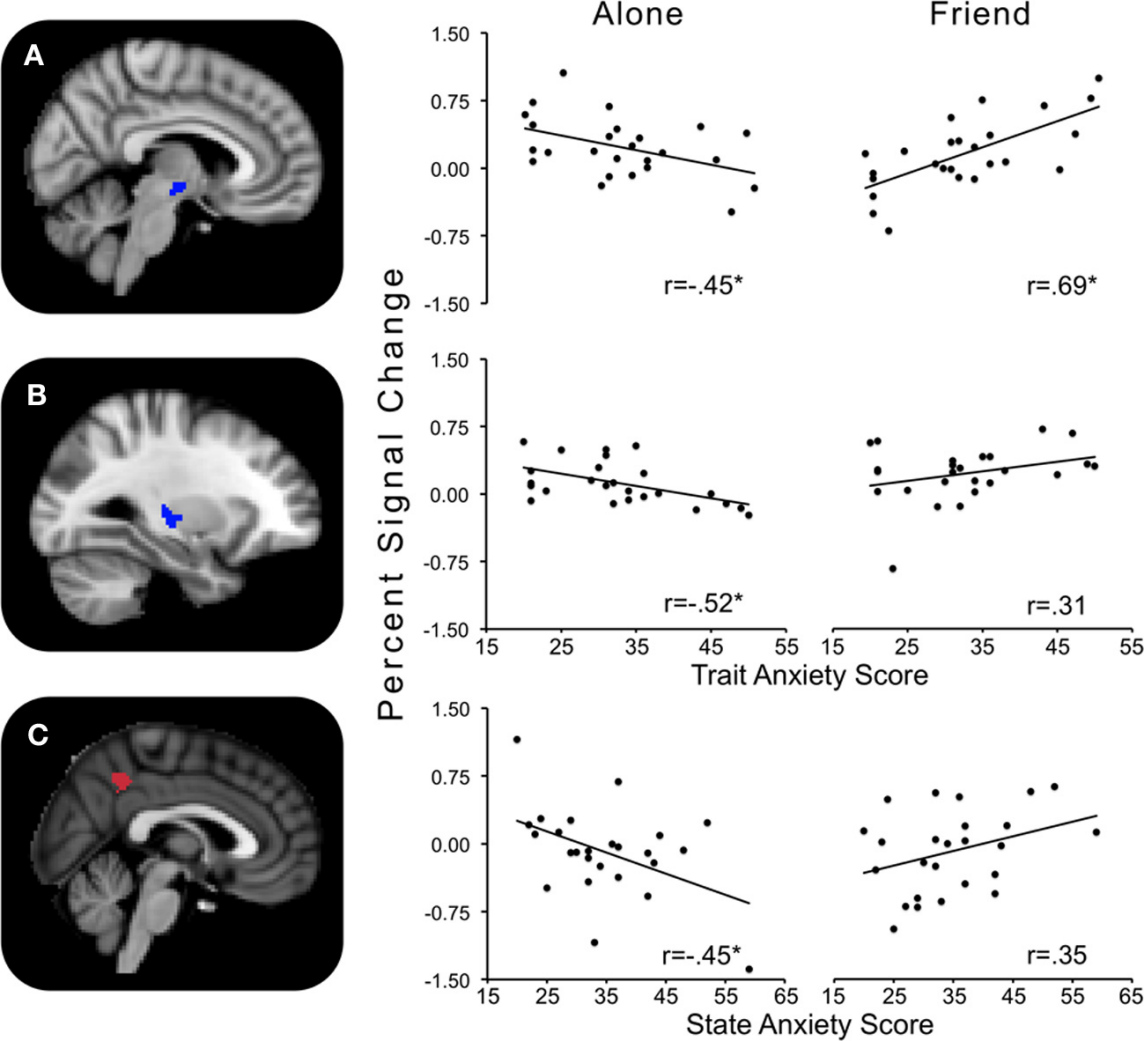
**Regions showing significant interactions between state or trait anxiety and Alone minus Friend conditions. (A)** Hypothalamus activity significantly related to trait anxiety; **(B)** left putamen activity significantly related to trait anxiety; **(C)** precuneus activity significantly related to state anxiety. ^*^p's < 0.05.

Trait anxiety significantly and negatively correlated with the Alone minus Friend contrast in two clusters, with one cluster peaking in the hypothalamus and extending to the substantia nigra, right pallidum, thalamus, insula, and putamen (Figure 4A), and another cluster peaking in the left putamen and extending to the left thalamus, insula, and pallidum (Figure 4B). These effects indicated that for those higher in trait anxiety, neural threat activation was decreased in the Alone condition relative to the Friend condition.

### Friend minus stranger

**Figure 5:**
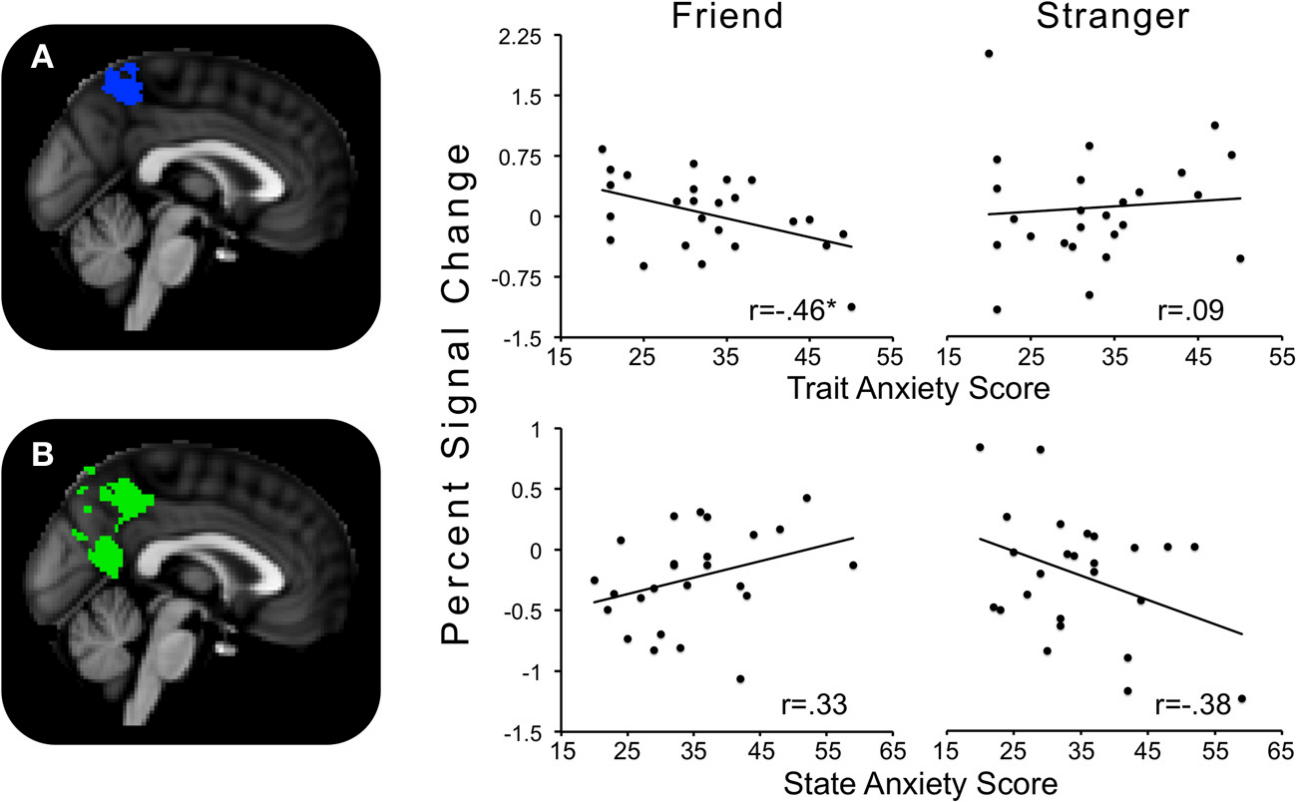
**Regions showing significant interactions between state or trait anxiety and Friend minus Stranger conditions. (A)** Precuneus activity significantly related to trait anxiety; **(B)** precuneus activity significantly related to state anxiety. ^*^p's < 0.05.

The contrast Friend minus Stranger resulted in one cluster of activity significantly and negatively correlated with trait anxiety. This cluster peaked in the left precuneus and extended to the left postcentral gyrus, indicating that for those with higher trait anxiety, neural threat activity was decreased in the Friend condition relative to the Stranger condition (Figure 5A).

### Interactions between handholding and state anxiety

State anxiety showed a significant negative correlation with threat-related brain activity in the Alone minus Friend contrast and a positive correlation in the Friend minus Stranger contrast (Table 2).

### Alone minus friend

One cluster in the Alone minus Friend contrast negatively correlated with state anxiety. This cluster peaked in the precuneus and extended to the PCC. This indicates that for those with higher state anxiety, neural activity was decreased in the Alone condition relative to the Friend condition (Figure 4C).

### Friend minus stranger

One cluster significantly positively correlated with state anxiety, peaking in the precuneus and extending into the PCC and lingual gyrus. In other words, for those higher in state anxiety, threat-related brain activity was higher in the Friend relative to the Stranger condition (Figure 5B).

### Individual threat and safety cues with state and trait anxiety

Because it is conceptually difficult to interpret correlations with fMRI contrast images, we considered the possibility that anxiety-related differences across handholding conditions were related to altered neural activity during the safety cues rather than during the threat cues. To explore this, we modeled the safety and threat cues independently with state and trait anxiety for each handholding condition. We saw no significant relationships between state or trait anxiety and neural activity during either the threat or safety periods in any condition, suggesting that people with higher state or trait anxiety did not have significantly different baseline or threat activity. We saw one exception: state anxiety was positively associated with neural activity during safety cues in the Stranger condition. This activity peaked in the left cuneal cortex and extended to the right cuneal cortex, the bilateral occipital pole, lateral occipital cortex, lingual gyrus, and cerebellum, and the right intracalcarine cortex.

## Discussion

Previously, we found that supportive social contact delivered via handholding reduced threat-related neural activity in the ACC, left superior frontal gyrus, and left supplementary motor cortex (Coan et al., 2013). Using the same sample, we examined how anxiety levels might interact with the presence or absence of supportive social contact to predict neural responses in the presence of a potential threat. Although trait and state anxiety were moderately correlated, their associations with active threats—both while alone and in a social context—were quite different. On the one hand, although trait anxiety was unrelated to the threat-safe contrast during supportive handholding, the same contrast was *negatively* associated with trait anxiety when participants were alone. This pattern was observed throughout the brain, implicating processes as diverse as self-focus, emotion, and working memory (e.g., precuneus, PCC, portions of the default mode network, cf. Maddock et al., 2003; Cavanna and Trimble, 2006; Zhao et al., 2007); motor preparation and coordination (e.g., precentral gyrus, supplementary motor cortex, and cerebellum, cf. Liotti et al., 2000; Critchley et al., 2004); and even visual attention (e.g., lateral occipital cortex and lingual gyrus, cf. Hopfinger et al., 2000; Murray and Wojciulik, 2004). While holding a friend's hand, trait anxiety corresponded with decreased brain activity mainly in the superior parietal lobule and frontal medial cortex, whereas higher state anxiety corresponded with *increased* brain activity in areas such as the PCC, frontal pole, and lateral occipital cortex. When holding a stranger's hand, neither trait nor state anxiety showed any association with brain activity. Close examination of these results suggested that trait anxiety indeed corresponded with smaller differences between threat and safety cues when a participant was alone in the scanner, relative to holding a stranger's or friend's hand.

These findings are consistent with the vigilance/disengagement model—that a curvilinear association between anxiety and neural output exists, such that moderate threats induce increased neural threat activity indicative of increased arousal and orientation to the threat, whereas strong threats induce decreased neural activity, signaling a disengagement or avoidance of the stimulus. Based on these and earlier results, we propose that social support can alter the perception threat—as well as the brain's multifaceted response to that threat—especially when the support is provided by a familiar friend. Moreover, the seemingly paradoxical impact of support on individuals high in trait anxiety may suggest some important clinical implications, for how anxiety is both understood and treated.

Previously, anxiety and related traits such as neuroticism have been characterized by increased reactivity to stress (Bolger and Schilling, 1991; Mroczek and Almeida, 2004). Accordingly, many studies employing neuroimaging have observed increased activity in threat-related brain regions in anxious individuals when anticipating an aversive stimulus (Canli et al., 2001; Simpson et al., 2001; Simmons et al., 2006; Haas et al., 2007). However, this finding has not been universal—in line with our findings, some have reported *decreased* neural activity in more anxious individuals (Kumari et al., 2007; Straube et al., 2009; Drabant et al., 2011).

We speculate that one key variable in resolving these discrepant findings may be how intensely the participant perceives the aversive stimulus during the anticipatory period. In general, studies employ an unchanging threat (e.g., a fixed level of shock) throughout the experiment. To examine the effect of varying the threat level on brain activity, Straube et al. (2009) employed a threat-of-shock paradigm in which participants underwent fMRI scanning while viewing cues indicating they might receive either no shock, mild shock, moderate shock, or strong shock, as subjectively rated by the participant prior to the scan. Participants retroactively reported their levels of state anxiety while anticipating each threat level. During moderate threat, positive correlations between anxiety and activity in the ventromedial prefrontal cortex (VMPFC) and vACC were found. Yet, during strong threat, these correlations became negative, while activity in the dorsal ACC, somatosensory cortex, motor cortex, and hippocampus showed positive correlations with anxiety.

While the Straube et al. (2009) study provides evidence of a curvilinear relationship that may help explain our findings, it is important to note that they assessed state anxiety *after* the fMRI scan and individually for each level of threat, whereas we looked at general measures of state and trait anxiety administered prior to the shock task. A more recent study measured levels of trait neuroticism prior to employing an fMRI paradigm in which level of shock was varied (Drabant et al., 2011). Results of this study showed a negative correlation between neuroticism and activity in the inferior frontal gyrus and insula in strong shock trials compared to moderate shock trials. The authors suggested that people high in neuroticism may switch to an avoidant processing strategy in the face of high threat, as would be predicted by the vigilance/disengagement model. Highly threatening stimuli seem to result in lower levels of threat-related brain activity in people with greater anxiety-related traits.

**Figure 6:**
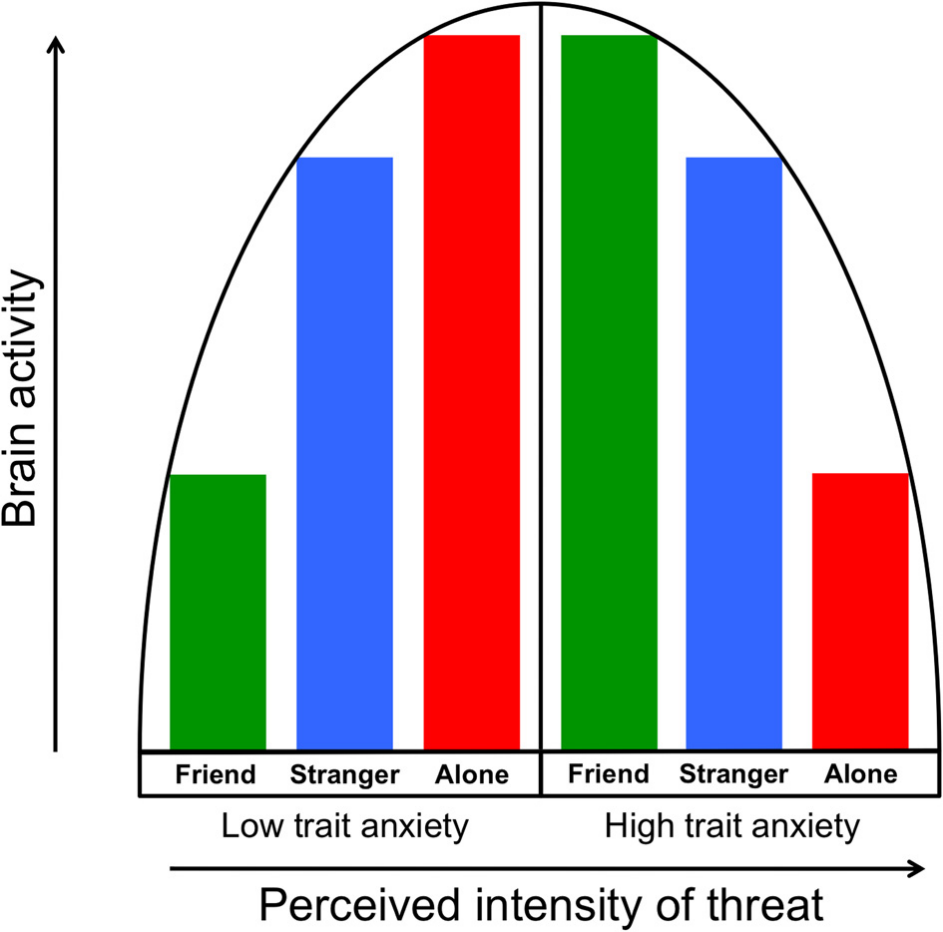
**The vigilance/disengagement model shows a curvilinear relationship between perception of threat intensity (in this case, a threat of shock) and neural threat-related activity**. As illustrated in the left half of the model, individuals with low trait anxiety show gradually increasing neural output as threat perception is increased via moderation by handholding condition. Those with higher trait anxiety, as illustrated in the right half of the model, start from a higher “baseline” of perceived intensity of threat, which results in gradually decreasing neural output as perceived threat intensity increases.

The vigilance/disengagement model we propose is consistent with an inverted U-shaped model of arousal outlined by Wilken et al. (2000) in which increasing arousal input (e.g., greater threat) also increases arousal output (e.g., physiological arousal) up to a point, at which output begins to decrease. They suggested that highly trait anxious individuals might be more aroused (and vigilant) at their “baseline,” such that severe stressors place them beyond the peak of the inverted U. Along these lines, we suggest first that the more anxious people in our sample may have perceived the threat of shock as a strongly threatening stimulus, leading to the regional neural deactivations we observed. Second, as we have previously documented the buffering effect of handholding on the brain's response to threat cues (Coan et al., 2006b, 2013), the administration of supportive handholding may have lowered the perception of threat in the highly anxious people to less intense levels (i.e., closer to the peak of the inverted U), resulting in increased neural activity. This model of the moderation of threat perception by social context is illustrated in Figure 6.

An important question to consider is whether our threat-of-shock paradigm could potentially be perceived as highly threatening. One limitation of our study is that we did not directly vary the level of shock, nor did we measure the subjective level of anxiety induced by the shock. However, we have several reasons to believe the nature of our shock paradigm is capable of inducing high levels of anticipatory anxiety. First, our threat cues did not indicate absolute certainty of shock; rather, we told the participants that the cues indicate a 17% chance of being shocked (and, indeed, we did shock them following 17% of the cues). This unpredictability may increase levels of negative affect and anxiety, as others have observed (Carlsson et al., 2006). Second, in contrast to other studies (Straube et al., 2009; Drabant et al., 2011), we did not shock the participants prior to entering the fMRI scanner; in other words, participants did not have a pre-formed expectation of the intensity of the shock until receiving a shock during the experiment. These factors, in combination with the tendency of people with high trait anxiety to interpret stimuli as more threatening than those with low trait anxiety (Mogg et al., 2000), suggest that our more anxious participants viewed the shock as a strong threat.

An alternative explanation for our findings is that high trait anxiety serves as a buffering factor to physiological arousal under times of high stress. Highly trait anxious people show blunted secretion of stress hormones such as cortisol, adrenocorticotropic hormone, epinephrine, norepinephrine, and prolactin during a social stress task (Jezova et al., 2004) and lower electrodermal responses during cognitive and affective stressors (Wilken et al., 2000). A previous study found that individuals with higher levels of neuroticism show less discomfort and smaller autonomic nervous system reactivity to a high intensity stressor (LeBlanc et al., 2004). Interestingly, in the same sample, more neurotic individuals reported greater discomfort to a mild or moderate stressor compared to less neurotic individuals, a behavioral finding that further suggests the vigilance/disengagement model (LeBlanc et al., 2003).

While our study focused on levels of anxiety in a subclinical sample, decreases in brain activity have been observed in individuals with a variety anxiety disorders in response to negative or threatening stimuli. For example, PTSD patients show less activity in the thalamus, parahippocampal gyrus, and parietal areas compared to controls when recalling negative emotional states (Lanius et al., 2003). A study using magnetoencephalography found early increased frontal activity in response to aversive pictures in PTSD patients relative to controls, followed by deactivations in parieto-occipital areas (Adenauer et al., 2010). People with generalized anxiety disorder (GAD) show increased early cortical activity followed by reduced reactivity, relative to healthy controls (Weinberg and Hajcak, 2011). These and similar findings have been posited to be a “vigilance-avoidance” pattern, in which rapid assessment of a threat is followed by attentional disengagement from the stimulus once it has been deemed dangerous (Mogg et al., 2004). This attentional disengagement, while it may decrease anxiety in the moment, maintains the anxiety disorder in the long run, as it prevents an individual from habituating to the feared stimuli. Our fMRI findings may have captured attentional disengagement, similar to that seen in anxiety disorders, in our more anxious participants. That social support moderated anxious responding during threat has important implications for the etiology and maintenance of anxiety disorders. It may be that anxiety-prone individuals are particularly vulnerable to experiencing a threat as highly threatening in the absence of social support, yet the lowered arousal resulting from disengagement may paradoxically reinforce the avoidance of social contact. Further research should assess this possibility.

In conclusion, we examined how anxiety relates to the neural response to threat under conditions of social support. We demonstrated that, when alone, participants with higher trait anxiety exhibited attenuated neural activity in several brain areas in response to a physically threatening stimulus, which we suggest is related to attentional disengagement. Upon receipt of social support via holding another person's hand, this effect largely disappeared or was reversed—brain activity in highly trait anxious people was indistinguishable from or slightly greater than that in less trait anxious people. These findings support a vigilance/disengagement model in which a curvilinear relationship between anxiety and threat results in decreased neural output past a certain threshold of threat intensity. That the provision of social support eliminated this effect suggests a role for supportive others in the treatment and prevention of anxiety disorders.

### Conflict of interest statement

The authors declare that the research was conducted in the absence of any commercial or financial relationships that could be construed as a potential conflict of interest.
